# A Novel Potential Positron Emission Tomography Imaging Agent for Vesicular Monoamine Transporter Type 2

**DOI:** 10.1371/journal.pone.0161295

**Published:** 2016-09-09

**Authors:** Zih-Rou Huang, Chia-Ling Tsai, Ya-Yao Huang, Chyng-Yann Shiue, Kai-Yuan Tzen, Ruoh-Fang Yen, Ling-Wei Hsin

**Affiliations:** 1 School of Pharmacy, College of Medicine, National Taiwan University, Taipei, Taiwan; 2 PET Center, Department of Nuclear Medicine, National Taiwan University Hospital, Taipei, Taiwan; 3 Molecular Imaging Center, National Taiwan University, Taipei, Taiwan; 4 PET Center, Department of Nuclear Medicine, Tri-Service General Hospital, Taipei, Taiwan; 5 Center for Innovative Therapeutics Discovery, National Taiwan University, Taipei, Taiwan; Biomedical Research Foundation, UNITED STATES

## Abstract

In the early 1990s, 9-(+)-^11^C-dihydrotetrabenazine (9-(+)-^11^C-DTBZ) was shown to be a useful positron emission tomography (PET) imaging agent for various neurodegenerative disorders. Here, we described the radiosynthesis and evaluation of the 9-(+)-^11^C-DTBZ analog, 10-(+)-^11^C-DTBZ, as a vesicular monoamine transporter 2 (VMAT2) imaging agent and compare it with 9-(+)-^11^C-DTBZ. 10-(+)-^11^C-DTBZ was obtained by ^11^C-MeI methylation with its 10 hydroxy precursor in the presence of 5 M NaOH. It had a slightly better average radiochemical yield of 35.3 ± 3.6% (decay-corrected to end of synthesis (EOS)) than did 9-(+)-^11^C-DTBZ (30.5 ± 2.3%). MicroPET studies showed that 10-(+)-^11^C-DTBZ had a striatum-to-cerebellum ratio of 3.74 ± 0.21 at 40 min post-injection, while the ratio of 9-(+)-^11^C-DTBZ was 2.50 ± 0.33. This indicated that 10-(+)-^11^C-DTBZ has a higher specific uptake in VMAT2-rich brain regions, and 10-(+)-^11^C-DTBZ may be a potential VMAT2 radioligand. Our experiment is the first study of 10-(+)-^11^C-DTBZ to include dynamic brain distribution in rat brains.

## Introduction

Vesicular monoamine transporter 2 (VMAT2), a member of the solute carrier family 18 with 12 transmembrane domains, is the protein responsible for transporting monoamine neurotransmitters (dopamine, norepinephrine, serotonin) into synaptic vesicles for subsequent storage and release [1,2]. VMAT2 abnormalities have been implicated in a variety of neurodegenerative disorders, including Parkinson’s and Huntington’s diseases [3,4]. VMAT2 also has been found to be highly expressed in human pancreas beta cells, which are related to diabetes [5–8], as well as in the central nervous system. However, the relationship between VMAT2 and the diseases mentioned previously or their underlying causes remains unclear. Positron emission tomography (PET) or single-photon emission computed tomography (SPECT) imaging of VMAT2 would further our understanding of its pathophysiology. PET is a non-invasive and highly sensitive technique that enables imaging of a live body using appropriate radiotracers and facilities. The resulting images could reflect the distribution and density of the target, which could provide valuable information regarding both the target and its relationship with diseases in the body. Given the strengths of PET, a specific PET tracer would be helpful in evaluating the body as well as the brain on a molecular level.

Currently, the radionuclides frequently used in PET are fluorine-18 and carbon-11, which have half-lives of 109 and 20 min, respectively. Although carbon-11 has a much shorter half-life, which limits its feasibility, it is still a useful radionuclide in clinical research because it allows multiple imaging sessions within one day. Therefore, studies of two or more protein targets in the same biological pathway are feasible after a short delay when using ^11^C-labeled radiotracers.

According to the literature, a VMAT2 PET radiotracer is primarily based on dihydrotetrabenazine (DTBZ) derivatives. The VMAT2 binding of DTBZ is stereospecific, and the (+)-enantiomer has a 1000-fold better binding affinity (*K*_i_ = 0.97 ± 0.48 nM) than does (-)-enantiomer (*K*_i_ = 2.2 ± 0.3 μM) [9–12]. Therefore, several structure activity relationship studies have been performed on the (+)-enantiomer. For ^18^F-labeled DTBZ derivatives, 9-^18^F-fluoropropyl-(+)-desmethyldihydrotetrabenazine (9-^18^F-FP-(+)-DTBZ) showed a good striatum-to-cerebellum ratio and is now in clinical study [13,14], while 9-^18^F-fluoroethyl-(+)-dihydrotetrabenazine (9-^18^F-FE-DTBZ) had a relatively poor striatum-to-cerebellum ratio, i.e. poor resolution [15]. For ^11^C-labeled DTBZ derivatives, ^11^C-methoxytetrabenazine (^11^C-MTBZ) showed a rapid accumulation in the brain followed by rapid clearance from all brain regions [16], while 9-(+)-^11^C-DTBZ has been investigated as a PET tracer for VMAT2 imaging since the early 1990s [17]. For example, 9-(+)-^11^C-DTBZ is now used in studies to differentiate types of dementia and evaluate their progression [18–27]. A 2014 study evaluated radiolabeled racemic DTBZ with carbon-11 in position 10, and the PET scan demonstrated symmetrical uptake in the striata (ST_R_/ST_L_ = 0.98 ± 0.05) of healthy rats [28]. As mentioned previously, VMAT2 binding of DTBZ is stereospecific, and therefore, our study included 10-(+)-^11^C-DTBZ. Here, we report on the synthesis of 10-(+)-^11^C-DTBZ and compare it with 9-(+)-^11^C-DTBZ as a VMAT2 PET imaging agent.

## Materials and Methods

### General

**Fig 1:**
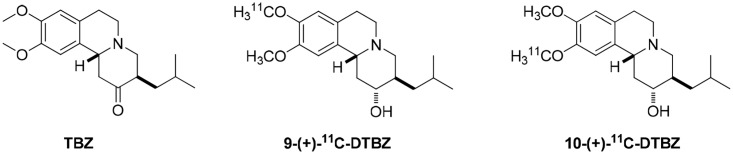
The chemical structure of TBZ and its derivatives.

Syntheses of the precursor to 9-(+)-^11^C-DTBZ were purchased from ABX. The precursor of 10-(+)-^11^C-DTBZ was synthesized according to Freyberg et al and can yield this precursor after hydrolysis [29]. (+)-DTBZ was prepared by reducing and demethylating tetrabenazine (TBZ) to obtain (+)-9-*O*-desmethyl-DTBZ or (+)-10-*O*-desmethyl-DTBZ. TBZ derivatives (Fig 1) were synthesized in the laboratory of the School of Pharmacy (National Taiwan University, Taipei, Taiwan). Sodium hydroxide was purchased from Sigma-Aldrich (St. Louis, MO, USA). Trifluoroacetic acid was purchased from Alfa Aesar (Ward Hill, MA, USA). Analytical reagent-grade reagents and solvents were purchased from Aldrich or Merck. The tC_18_ Sep-Pak and Sep-Pak Light QMA cartridges were acquired from Waters Chromatography Division, Millipore Corporation.

HPLC analysis was performed with a Waters HPLC system equipped with both UV (280 nm) and radioactivity detectors.

### Animal preparation

Rat studies were performed using 250–330 g male Sprague Dawley rats (BioLASCO Taiwan Co., Ltd.). All animal studies were reviewed and approved by the Institutional Animal Care and Use Committee. This work was approved by the Laboratory Animal Center of National Taiwan University College of Medicine. The animals were housed and handled according to institutional guidelines. All animals were starved overnight prior to the experiment. On the day of the study, rats were anesthetized using 5.0% isoflurane. Each rat was positioned on the scanner bed, and anesthesia was applied using a nose cone. A transmission scan was acquired. A radiotracer (0.8–1.3 mCi) was injected intravenously into the tail vein of the rat. Isoflurane was reduced and maintained at 2.0% following injection.

### Radiochemistry

**Fig 2:**
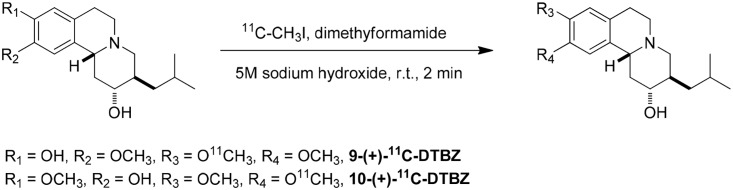
Radiochemical synthesis of 9-(+)-^11^C-DTBZ and 10-(+)-^11^C-DTBZ.

All steps (Fig 2) up to sterile filtration of the final product solution were performed using the integrated functions of the GE TRACERlab FXc synthesis system. The reaction of gaseous ^11^C-methyl iodide with (2*R*, 3*R*, 11b*R*)-10-*O*-desmethyldihydrotetrabenazine or (2*R*, 3*R*, 11b*R*)-9-*O*-desmethyldihydrotetrabenazine took place in the FXc reaction vessel. The vessel was charged with 2.0 mg of precursor, 15 μL of 5 M NaOH and 400 μL of dimethylformamide. ^11^C-Methyl iodide was extracted from the methyl iodide synthesis unit and introduced into the reaction vessel at a fixed flow rate of 15 mL/min for 3 min. The flow was then stopped, and the solution was stirred for an additional 2 min. The reaction mixture was diluted with 0.6 mL of ethanol/50 mM of NH_4_OAc adjusted to pH = 4.5 by acetic acid (10:90); injected into a preparatory HPLC column (Waters XTerra Prep, 10 μm, 10 x 150 mm); and eluted with 10/90 ethanol/50 mM of NH_4_OAc adjusted to pH = 4.5 by acetic acid at 6 mL/min. The product fraction was collected at 9.5 min, diluted with isotonic saline and passed through a 0.22-μm sterilizing filter into a sterile 10-mL multi-dose vial.

### Quality control

Chemical and radiochemical impurities were detected using HPLC. For a quality assessment with analytical HPLC analysis, a reversed-phase C_18_ column (Phenomenex Gemini, 5 μm, 4.6 x 250 mm) with acetonitrile:50 mM of NH_4_OAc adjusted to pH = 4.5 by acetic acid (20:80) as the eluent was used with a flow rate of 1 mL/min. The retention times of 9-(+)-^11^C-DTBZ and 10-(+)-^11^C-DTBZ were 9.8 and 10.5 min, respectively, and both radiotracers were further confirmed with authentic standards for radiochemical identities.

### MicroPET imaging

For the animal study, fasting male Sprague Dawley rats (n = 3) were used. The rats had free access to water for 12 hours prior to the experiment and were then injected with a bolus of 0.8–1.3 mCi of radiotracer. A small-animal Argus PET/CT scanner was used to produce dynamic sinograms for 90 min with 4 x 15 sec, 2 x 30 sec, 4 x 60 sec, 3 x 180 sec, 5 x 300 sec, 3 x 600 sec and 1 x 1200 sec frames.

Image analyses of the rat brain were performed with PMOD, version 3.7 (Sciffer, PMOD Technologies Ltd.). First, rat brain images were aligned to a standard template of a rat brain using PMOD’s rigid matching tool. The region of interests of each rat brain were then manually defined and expressed as standard uptake values (SUVs). The specific uptake ratios (SURs) of the striata were expressed as striatum/ cerebellum.

### Blocking studies

The blocking studies were carried out by pretreating the rats (250–275 g, n = 3) with unlabeled DTBZ for 2 h (2 mg/kg; intravenous bolus) prior to 10-(+)-^11^C-DTBZ or 9-(+)-^11^C-DTBZ (0.9–1.1 mCi) intravenous administration. The dynamic sinograms were obtained by using a small-animal Argus PET/CT scanner for 50 min with 4 x 15 sec, 2 x 30 sec, 4 x 60 sec, 3 x 180 sec, 5 x 300 sec, and 1 x 600 sec frames.

## Results and Discussion

PET tracers designed to target specific neurochemical processes offer new possibilities for improving the differentiation of various central nervous system-related disorders, such as dementia [2]. Therefore, because of the physiological importance of VMAT2, we proposed to synthesize 10-(+)-^11^C-DTBZ, the regioisomer of 9-(+)-^11^C-DTBZ, which has been widely used for VMAT2 imaging, and we performed a comparative in vivo evaluation of both radiotracers.

We first attempted to use a (+)-9,10-dihydroxy precursor to obtain either 9-(+)-^11^C-DTBZ or 10-(+)-^11^C-DTBZ. With the adjustment of the base, 9-(+)-^11^C-DTBZ and 10-(+)-^11^C-DTBZ were obtained at a 5:2 ratio. However, an unknown radioactive product was produced in addition to 9,10-(+)-^11^C-DTBZ, and we could not isolate the individual 9-(+)-^11^C-DTBZ or 10-(+)-^11^C-DTBZ with the separation system that was available.

**Fig 3:**
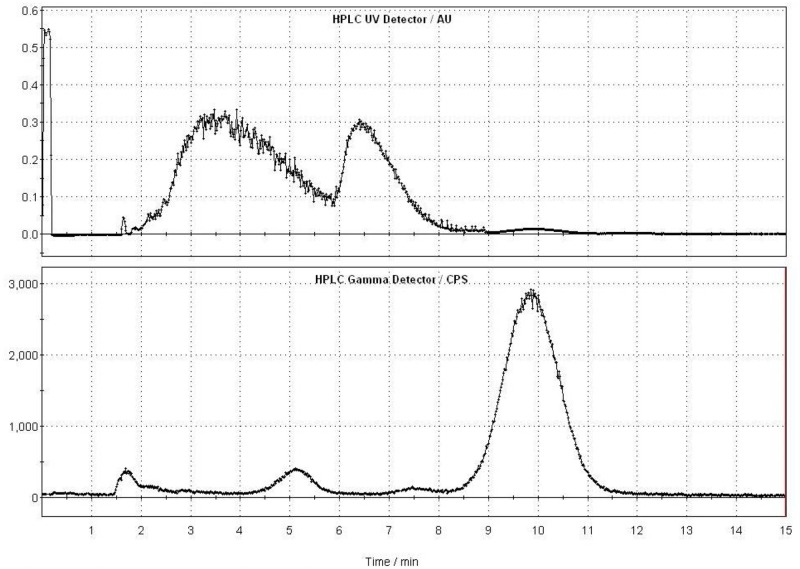
Semi-preparative HPLC purification chromatograms of 10-(+)-^11^C-DTBZ (Waters XTerra, 10 x 150 mm, 50 mM NH_4_OAc adjusted to pH = 4.5 with acetic acid/ethanol, 90/10, v/v, 6 mL/min), panel A: UV detection at 280 nm, panel B: radioactivity. The product fraction was collected at 9.6 to 10.6 min.

**Fig 4:**
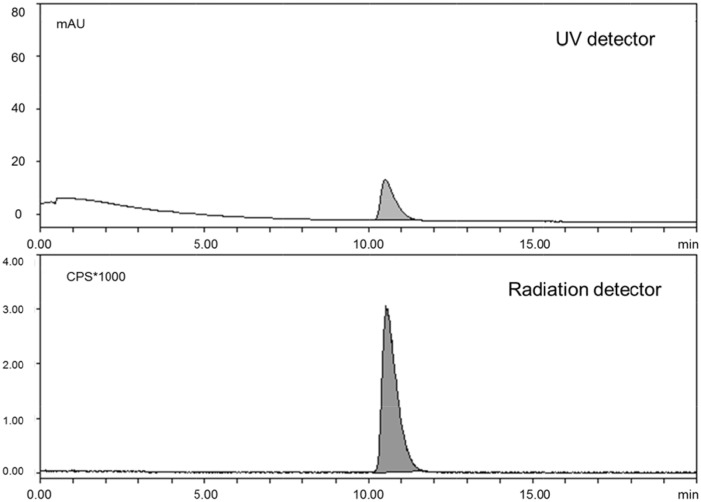
HPLC chromatograms of purified 10-(+)-^11^C-DTBZ and DTBZ. The chromatograms were obtained using a radiation detector and a UV detector, respectively, under the same analytical HPLC conditions.

Briefly, ^11^C-CO_2_ was produced by a ^14^N(p,α)^11^C reaction in a PETtrace cyclotron and transferred to a TRACERlab F_Xc_ module to produce a ^11^C-CH_4_ via H_2_(g)/Ni reduction. Following gas halogenations of ^11^C-CH_4_, the obtained ^11^C-methyl iodide was trapped in a vessel containing either the base-protonated precursor (+)-9-demethyl-DTBZ for 9-(+)-^11^C-DTBZ or (+)-10-demethyl-DTBZ for 10-(+)-^11^C-DTBZ. After ^11^C-methylation, semi-preparative HPLC was performed to purify the labeled product (Fig 3). The radiochemical yields (EOS) of 9-(+)-^11^C-DTBZ and 10-(+)-^11^C-DTBZ were 30.5 ± 2.3% (n = 3) and 35.3 ± 3.6% (n = 3), respectively, with > 99% radiochemical purity (Fig 4) and 29 min of synthesis time. Quality control tests were then performed. The chemical purities of both radioligands were greater than 95% and were radiochemically stable for at least 1 h. The specific activities of 9-(+)-^11^C-DTBZ and 10-(+)-^11^C-DTBZ were 63 ± 4 (n = 4) and 82 ± 3 (n = 6) GBq/μmol (end of bombardment), respectively.

**Fig 5:**
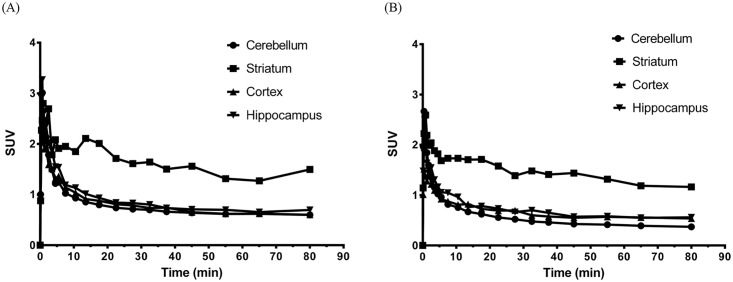
Time-activity curves of (A) 9-(+)-^11^C-DTBZ and (B) 10-(+)-^11^C-DTBZ in normal Sprague Dawley rat brains. PET data were collected for 90 min. Regions of interest (cerebellum, striatum, cortex and hippocampus) were identified according to the stereotaxic atlas, and the radioactivities were plotted against the time post-injection. Each value represents the mean ± SD (n = 3).

**Fig 6:**
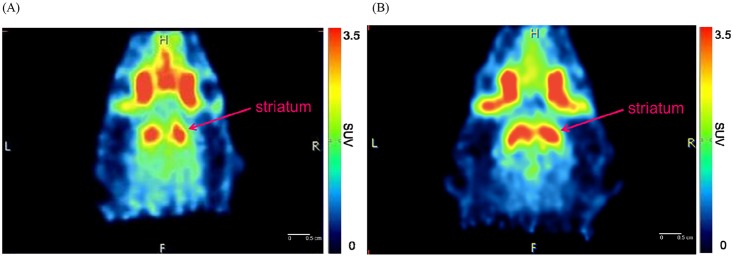
PET images of (A) 9-(+)-^11^C-DTBZ and (B) 10-(+)-^11^C-DTBZ are coronal slices at the level of the striatum and cerebellum and represent summations of 8–90 min of emission data. Each value represents the mean ± SD (n = 3).

**Fig 7:**
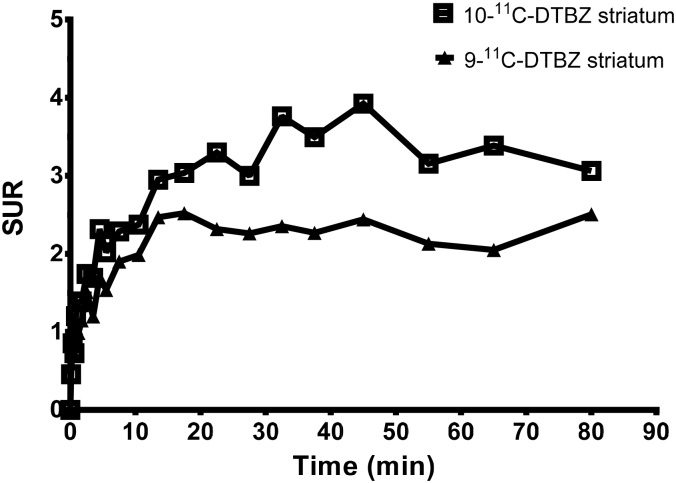
The SUR comparison of 9-(+)-^11^C-DTBZ and 10-(+)-^11^C-DTBZ in a rat brain.

**Fig 8:**
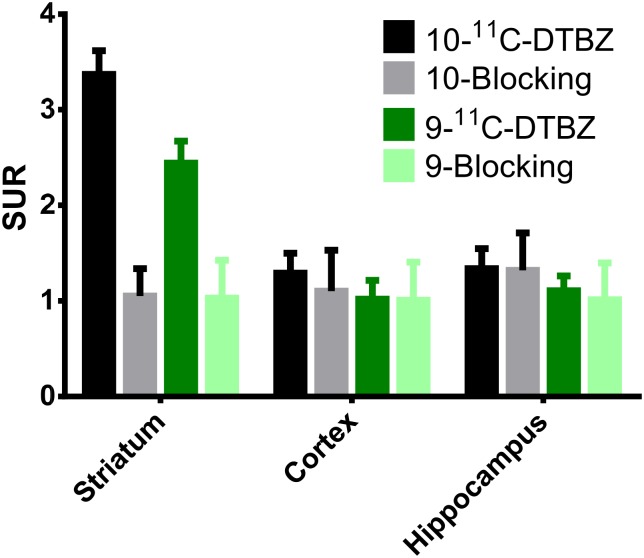
The regional rat brain uptake of 9-(+)-^11^C-DTBZ and 10-(+)-^11^C-DTBZ in the control and pretreatment (blocking with 2 mg/kg of unlabeled DTBZ) groups at 50 min. Each value represents the mean ± SD (n = 3).

An eXplore Vista-DR (GE) small-animal PET scanner was used for the experiment on Sprague Dawley rats. A series of PET studies was performed on the rats with 0.8–1.3 mCi of each radioligand administered via the tail vein, and dynamic PET images were acquired for 90 min. Reconstructed images were aligned with a standard rat brain regional template, and time-activity curves were obtained for the cerebellum, striatum, cortex and hippocampus. Fig 5(A) and 5(B) show the time-activity curves of 9-(+)-^11^C-DTBZ and 10-(+)-^11^C-DTBZ in different rat brain regions. These microPET images of rat brains showed a high level of uptake in the striatal regions but low uptake in the cerebellum. Because the egress of radioactivity of both radioligands from various brain regions was very rapid, good differentiation of the striatum from the cerebellum was visible between 8 and 90 min after injection (Fig 6). The SURs of 9-(+)-^11^C-DTBZ and 10-(+)-^11^C-DTBZ in brain regions at different time points are shown in Fig 7. Compared to the SUR of 9-(+)-^11^C-DTBZ (2.50 ±0.33, Table 1, Fig 7), the SUR of 10-(+)-^11^C-DTBZ peaked (3.74 ± 0.21) at 40 min post-injection. The in vivo binding specificities of 9-(+)-^11^C-DTBZ and 10-(+)-^11^C-DTBZ for VMAT2 were investigated by performing blocking studies on rats. Healthy rats were pretreated with unlabeled DTBZ (2 mg/kg; intravenous injection) for 2 h, administered 9-(+)-^11^C-DTBZ or 10-(+)-^11^C-DTBZ intravenous and then subjected to a PET scan over a period of 50 min. The PET results showed that the regional brain uptake of 9-(+)-^11^C-DTBZ and 10-(+)-^11^C-DTBZ in the striatum in the pretreatment group were blocked by the unlabeled DTBZ, and their SURs were similar to that of the cerebellum (Fig 8). Particularly in the striatum, the uptake of 10-(+)-^11^C-DTBZ was obviously prevented compared to the control group, and the striatum/cerebellum SUR decreased from 3.74 (control group) to 1.08 (pretreatment group). However, the uptake of 9-(+)-^11^C-DTBZ was obviously prevented compared with the control group, and the striatum/cerebellum SUR decreased from 2.50 (control group) to 1.03 (pretreatment group). Therefore, the binding specificities of 10-(+)-^11^C-DTBZ and 9-(+)-^11^C-DTBZ for VMAT2 were confirmed. In our experiment, the transient equilibrium of 10-(+)-^11^C-DTBZ in rat brains was determined by a dynamic brain distribution study. In addition to 9-(+)-^11^C-DTBZ, the brain uptake and clearance kinetics of 10-(+)-^11^C-DTBZ were similar to those of previously studied radioligands, such as ^11^C-tetrabenazine (^11^C-TBZ), ^11^C-methoxytetrabenazine (^11^C-MTBZ), 9-^18^F-fluoroethyl-(+)-dihydrotetrabenazine (9-^18^F-FE-DTBZ) and ^18^F-9-fluoropropyl-(+)-dihydrotetrabenazine (9-^18^F-FP-DTBZ) [15,30,31]. Our results also indicate that biodistribution studies with 10-(+)-^11^C-DTBZ have good reproducibility.

**Table 1:** Synthesis and microPET imaging data of 9-(+)-^11^C-DTBZ and 10-(+)-^11^C-DTBZ.

	9-(+)-^11^C-DTBZ	10-(+)-^11^C-DTBZ
Synthesis time (min)	35 ± 4	34 ± 5
Radiochemical yield (%, EOS)	30.5 ± 2.3	35.3 ± 3.6
Specific activity (GBq/μmol)	63 ± 4	82 ± 3
Striatum (SUV)	1.75 ± 0.07	1.65 ± 0.10
Cerebellum (SUV)	0.70 ± 0.45	0.44 ± 0.30
ST/CB	2.50 ± 0.33	3.74 ± 0.21

Each value represents the mean ± SD (n = 3).

Abbreviations: DTBZ, dihydrotetrabenazine; EOS, end of synthesis; SUV, standardized uptake value; ST, striatum; CB, cerebellum.

Taken together, these results suggest that 10-(+)-^11^C-DTBZ may have favorable properties as a VMAT2 PET radiotracer. Additional research, including metabolism profiles, is underway for future clinical and preclinical studies. To our knowledge, this is the first investigation to demonstrate the region-selective effects of binding to 10-(+)-^11^C-DTBZ in the rat brain.

## Conclusion

In this study, a novel carbon-11-labeled DTBZ derivative, 10-(+)-^11^C-DTBZ, was synthesized and evaluated in vivo using microPET imaging. MicroPET studies demonstrated that 10-(+)-^11^C-DTBZ had good brain penetration and high specificity for brain VMAT2. Based on the microPET data, the striatum-to-cerebellum ratio peaked (3.74 ±0.21, n = 3) at 40 min post-injection, which was higher than the ratio for 9-(+)-^11^C-DTBZ (2.50 ±0.33, n = 3). From our preliminary results, 10-(+)-^11^C-DTBZ could be a potential PET imaging agent for VMAT2 and could be used in the diagnosis and monitoring of VMAT2-related disorders, such as Parkinson’s disease and diabetes.
